# What Did Chaplains Do During the Covid Pandemic? An International Survey

**DOI:** 10.1177/1542305021992039

**Published:** 2021-03-17

**Authors:** Austyn Snowden

**Affiliations:** Edinburgh Napier University, Edinburgh, UK

**Keywords:** Covid 19, pandemic, chaplain, international, spiritual care

## Abstract

Chaplains’ unique contribution is to healthcare is to respond to the spiritual, religious and pastoral needs of patients and staff. This is their sole purpose, to provide a presence and space to meet individual need and promote healing, even when cure isn’t possible. Their value is priceless to families in desperate times. However, despite growing evidence for their impact, chaplains are commonly undervalued and misunderstood by their organisations, and the global pandemic revealed the consequences of this confusion. Whilst some chaplains were applauded as heroes along with their fellow health colleagues, others were seen as little more than an infection risk. A survey was designed to capture and learn from the full range of chaplain experiences of the impact of the pandemic across the globe. In June 2020, 1657 chaplains responded from 36 countries. They all experienced considerable disruption to their usual practice, with enforced social distancing having the biggest impact. Out of necessity they embraced technology to maintain contact with patients and families, and shifted focus of their support to staff. Whilst some chaplains were viewed as essential employees by their organisations, most were not. Despite the majority thinking that their organisations understood what they did, chaplains themselves were neither clear or unclear about their role during and post pandemic. More surprisingly, they felt similarly unclear about their role *before* the pandemic. This paper concludes that in general chaplains lack leadership skills, and confusion about their role will persist until this changes.

## Introduction

Chaplains’ unique contribution to health and social care is their ability to respond to the spiritual, religious and pastoral needs of patients and staff. This is their sole purpose, to respond in a confidential, timely manner without the constraints or restrictions that other professionals may experience in their roles (Carey et al., 2016). They can take time and provide a presence and space to meet spiritual need. Chaplains promote healing, even when cure isn’t possible (Massey et al., 2015)

However, despite the growing evidence for their impact (Kestenbaum et al., 2017; Macdonald, 2017), chaplains in healthcare remain widely misunderstood, undervalued and underused (Snowden et al., 2020). Never has this been more starkly realised than during the recent Covid-19 pandemic. Whilst some reports suggested chaplains had been highly valued and effectively deployed during this time (Cawley & Precey, 2020), there were other anecdotal reports of chaplains being asked not to report to work at all by their employers (Ganiel, 2020). The pandemic thus inadvertently offered an opportunity to systematically examine how different health systems around the world understood, valued and used their chaplains in a ‘spiritual emergency’. This study was designed to capture and analyse the full range of chaplain experience in an international sample of healthcare chaplains.

## Background

The Covid-19 pandemic caused by the corona virus confronted people worldwide with a crisis on an unprecedented scale. Countries closed their borders and restricted movement in attempts to contain the spread of infection. Health systems around the world prepared to be overrun with cases as the most vulnerable people in society bore the brunt of the virus. The social and economic impact will not become clear for years. However, one unintended outcome of the pandemic was widespread recognition of the value of health workers (Winiger, 2020). All were universally honoured as ‘heroes’: the doctors, nurses, cleaners – all those who ensured in particular that the overburdened ICUs would be able to provide their care (Schutz & Shattell, 2020).

This focus on the ICUs was unavoidable given the serious impact on many patients. As of end of June 2020, half a million people had died from the virus worldwide, and at the end of September that figure had risen to 1 million (BBC,2020). Covid-19 patients and their relatives had to learn to cope with isolation measures as healthcare became dominated by infection-controlled ICU’s and Covid-19 wards. Patients with different disorders and nursing home residents were forgotten (Williams, 2020), and social distancing became the ‘new normal’(Rodner, 2020). Person centred care appeared to be temporarily superseded by a utilitarian approach in which the quelling of the virus became the sole aim (Trepanier, 2020).

One of the major casualties of this agenda was the spiritual dimension, precisely the dimension represented by chaplains (Kelly, 2013). For many patients and their families, coping with illness, traumatic events and facing the end of life are not just medical issues, they are deeply spiritual issues (Fitchett et al., 2020). However, patients, staff and relatives had become isolated from each other as a consequence of the measures designed to save them. To do their jobs, chaplains had to become creative to militate against any barriers put in their way. This research was designed to capture that creativity as well as other changes to practice whilst still fresh in the mind of participants.

In short, it was reasonable to assume that chaplains around the world would have been substantially challenged during the pandemic. Where chaplains had overcome these challenges, there would be transferable learning in that some of the novel ways of being a chaplain could be worth maintaining post pandemic. At the opposite extreme, some chaplains appear to have been excluded or marginalised, suggesting a deep misunderstanding of the value of spiritual care in uncertain times. Again, if true, it would be important to unearth such instances to better understand why this may have happened to prevent it happening in the future. For better or worse, healthcare chaplains*^1^ around the world have reacted to best support their local populations. The overarching aim of this international study was to understand how and to whom chaplains delivered spiritual care during the first, acute phase of the global Covid-19 pandemic.

## Method

### Aim

To understand how chaplains delivered spiritual care during the Covid-19 global pandemic.

### Objectives

1. Describe how healthcare chaplains delivered spiritual care during the pandemic.
2. Identify and analyse key changes to usual practice

### Design

International cross-sectional survey design

### Population

#### Inclusion Criteria

Healthcare Chaplains* currently employed to deliver spiritual care in healthcare settings, such as hospitals, hospices, primary care, community care and nursing homes. Over 18 years. Able to read English, although responses to free text can be given in any language. Access to electronic device connected to internet.

#### Exclusion Criteria

Under 18 years. Volunteers, or other healthcare workers (nurses, doctors, allied health professionals) not employed as Healthcare Chaplain or equivalent*.

### Data

The survey was constructed and collated in NOVI™, a secure, encrypted and password protected survey platform hosted at Edinburgh Napier University, Scotland. A link to the survey was posted online in various social media outlets and hosted by specialist chaplain organisations. It was sent to email networks of healthcare chaplains in Europe, USA and Australia, with reminder follow-ups after one week. Collection ran in May and June 2020.

**Table 1.** Participants to the Chaplain Covid-19 Survey.

Australia	202
Austria	9
Belgium	131
Canada	40
Chile	2
Colombia	3
Cyprus	2
Czech Republic	9
Denmark	1
Estonia	5
Finland	13
France	9
Germany	89
Hong Kong	1
Hungary	4
India	3
Indonesia	1
Ireland	22
Israel	4
Italy	5
Kenya	1
Latvia	1
Luxembourg	9
Malta	1
Netherlands	173
Norway	16
Philippi	4
Portugal	2
Romania	1
Spain	4
Sweden	15
Switzerland	31
UK	109
Ukraine	1
USA	688

### Survey

The survey was designed and reviewed over a two-week iterative cycle of testing and refining on a range of platforms and devices with the help of chaplain colleagues in Australia, USA and Europe. Because of the exploratory nature of the research, the survey was designed to obtain a broad mix of quantitative and qualitative data with the emphasis on encouraging free text responses, but without being too onerous for participants to complete. The final version generated relevant data, was reportedly easy to understand for participants, and took on average between 12 to 15 minutes to complete. The key questions are in Box 1.

Box 1. Survey Items and Their Relationship to the Study Objectives and Research Questions.

**Table:** 

Survey items and their relationship to the study objectives	
Survey question	Associated Objective
Continent, country, age, gender, faith, years as chaplain, qualification, professional association (Table 2)	*Describe how healthcare chaplains delivered spiritual care during the pandemic.*
Which setting do you usually work in?	
Which setting did you work in during the pandemic?	
When/how were you asked to provide spiritual care?	
Did you have access to COVID-19 patients?	
Did you have access to NON COVID-19 patients?	
Did staff other than the chaplains deliver spiritual care?	
Did you have regular supervision, intervision, or other form of regular group reflection?	
Did you feel that you could have been better deployed during the pandemic?	*Identify the key changes to usual practice and examine their impact on patients, chaplains, staff and culture.*
Did your organisation understand the contribution you could offer to COVID-19 patients?	
How clear was your role to you during pandemic?	
How anxious/afraid were you?	
How have you taken care of yourself since the beginning of the pandemic?	
Which (if any) of the following issues had the greatest impact on you during the pandemic?	
Was your professional association or faith/belief group supportive?	
*What has changed as a function of the pandemic?*	*Free text questions. Discussed in subsequent papers.*
*What has been lost as a function of the pandemic?*	
*What has been gained as a function of the pandemic?*	
*What new innovation should be kept after the pandemic?*

### Ethics

Permission to undertake the study was given by School of Divinity Ethics Committee at KU Leuven University. Each chaplain was emailed a participant information sheet detailing the scope of the study and what to expect should they choose to participate in the survey. They were also shown further information on data protection, privacy and data management when they opened the survey online. It was made clear that participation was voluntary, that no harm would come as a consequence of *not* participating, and that participants could withdraw any time they wanted. No identifying information was requested or collected, and every participant was given the opportunity to ask any questions they may have had. Each participant completed a consent form and confirmed they had read and understood all participant information online prior to completing the survey.

## Results: Demographics

The survey was returned by 1657 chaplains in May and June 2020. Data were downloaded as excel spreadsheet. Free text entries were extracted and imported into NVivo™. The remaining data file was imported into SPSS, coded and cleaned by checking for outliers (Lund & Lund, 2017). This process revealed two participants declaring their age to be over 100 years, and an additional two declaring over 100 years of working as a chaplain. These four entries were double checked for other anomalies and three were clearly nonsensical, ticking the most extreme response in every possible case. These entries were removed from further analysis. The final outlier had declared ‘222’ years of being a chaplain, but the entry was otherwise clearly written by a chaplain because of the authentic free text responses. This entry was therefore retained, but ‘time as chaplain’ amended to ‘22’ years as typo was the most likely explanation for this error. This process left a complete dataset of 1654.

Responses came from 36 countries spanning six continents (see Table 1). The majority came from North America (n = 730), Europe (n = 666), Australia (n = 202) with Asia (n = 12), South America (n = 7) and Africa (n = 1) making the rest. Thirty-six respondents didn’t state their continent. Mean (SD) age was 54 (11.65) years, with mean 13.55 (10.22) years as a healthcare chaplain. The sample was predominantly female (n = 920), with males (n = 660), other (n = 9) and 20 preferring not to say. Most had Masters level degree by way of highest qualification (n = 1040), followed by 235 reporting doctoral level education and a similar number reporting bachelor degree (n = 234). The remainder were high school (n = 23) or ‘other’ (n = 99).

Christian Protestants were the largest faith group (n = 924), followed by Christian Catholics (n = 412), Jew (n = 64), Humanist (n = 46), Buddhist (n = 18), Muslim (n = 12) and Hindu (n = 6). Ninety-eight did not respond and 74 declared ‘other’ as main religion or faith. Most (n = 1187) belonged to a professional association, although 323 did not; and most worked as part of a team (n = 1232) as opposed to working alone (n = 266). Table 2 summarises these demographics according to continent. Where cells contain less than 10 participants, no value is reported to protect participant anonymity. Participants from Africa, Asia and South America have therefore been omitted from this element of descriptive analysis for this reason. Their free text comments are included in subsequent papers.

**Table 2.** Demographics summarised by continent.

Continent	Age (SD) yrs	Years (SD) as chaplain	Gender		Main religion or faith		Highest qualification		Team or alone		Professional association	
			n		n		n		n		n	
North	58 (13.1)	14 (10.9)	Male	308	Protestant	481	PhD	147	Team	590	Yes	562
America			Female	391	Catholic	105	Masters	528	Alone	84	No	114
					Jew	56	Bachelor	27				
					Humanist	<10	High School	<10				
					Buddhist	<10						
												
Europe	52.5 (10.8)	14 (9.7)	Male	259	Protestant	325	PhD	66	Team	466	Yes	499
			Female	391	Catholic	234	Masters	422	Alone	137	No	113
					Jew	<10	Bachelor	119				
					Humanist	40	High School	12				
					Buddhist	<10						
												
Australia	57.8 (9.1)	9.5 (7.7)	Male	70	Protestant	105	PhD	11	Team	148	Yes	96
			Female	124	Catholic	63	Masters	70	Alone	39	No	92
					Jew	<10	Bachelor	87				
					Humanist	<10	High School	<10				
					Buddhist	<10						

## Results: Survey Responses

### Which Setting Do You Usually Work In? Which Setting Did You Work in During the Pandemic?

Most chaplains worked in the same place during the pandemic as they had done previously. The largest displacement was for 14 chaplains, who moved from hospital to ‘other’ (eg parish, military, community, prison, private practice, palliative care, school, primary care (general practice), continuing care). However, this was against 780 chaplains who usually worked in hospital and stayed there during the pandemic.

### When or How Often Were You Asked to Provide Spiritual Care?

**Figure 1.**
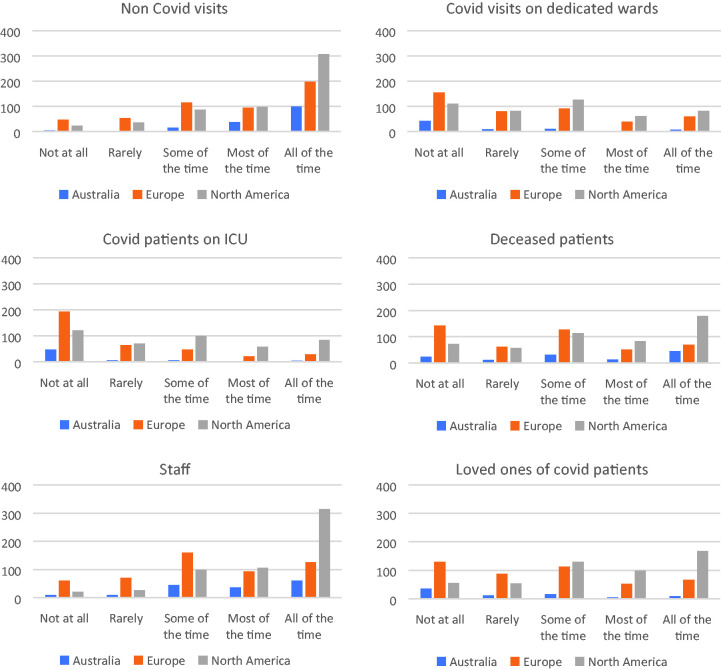
‘When were you asked to provide spiritual care?’ Chaplain self-described activity in relation to caring for patients, families and staff.

The six bar charts in Figure 1 show how chaplain time was used during the Covid-19 outbreak across the world. The x axes represent the same Likert scale going from ‘not at all’ to ‘all the time’, and the y axes represent the number of respondents.

Overall, they show that interactions with *non*-Covid-19 patients and staff were in the majority during this period, although there was some engagement with Covid-19 patients and families too. There may be a difference between the continents in the way chaplains interacted with Covid-19 patients in ICU, with this appearing to be a more common occurrence for American chaplains than either European or Australian chaplains. On the whole however, response patterns were relatively similar around the world.

### Did You Have Access to Covid-19 Patients/Non Covid-19 Patients?

**Figure 2.**
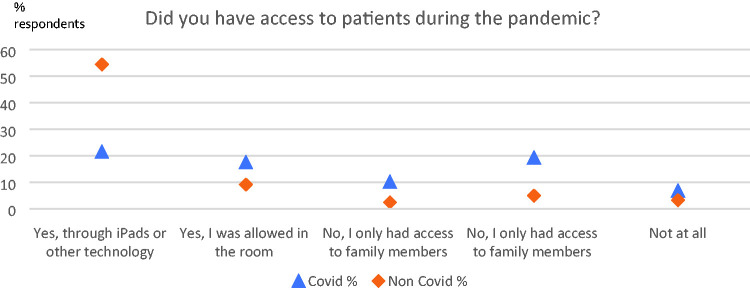
Types of access to patients and families during pandemic according to whether patients Covid-19 or non-Covid-19, (N = 1179).

Figure 2 shows the breakdown of the types of work practices undertaken according to the Covid-19 status of the patient. The high proportion of time spent supporting patients *without* Covid-19 but *still using technology* is of note. Free text comments described chaplains working differently, sometimes meeting patients outside wards for example. Others stated lack of PPE prevented them seeing either Covid-19 or non-Covid-19 patients, and more spoke about the fluctuating nature of the situation. Absence of baseline measures meant any inference about change needs to be treated with caution, but given that using technology to access patients was not the norm prior to pandemic, then Figure 2 appears to indicate there was a substantial switch to using technology to care for all patients, whether Covid-19 or not.

### Did You Have Regular Supervision, Intervision, or Other Regular Group Reflection?

**Figure 3.**
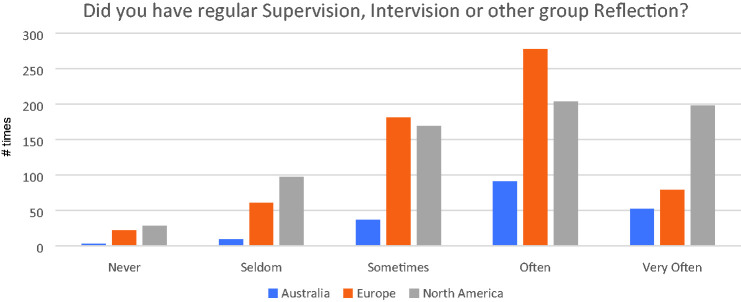
Frequency of supervision during pandemic.

Figure 3 shows that frequency of supervision during the pandemic remained reasonably high in all three continents, although again without baseline it is not possible to comment on whether this represents a change or not.

### Did Staff Other Than the Chaplain Do Spiritual Care?

Table 3 shows staff other than chaplains doing spiritual care was common around the world. Responses were expanded upon in free text comments by 550 participants. Elaborations were framed by two diametrically opposed perspectives: ‘spiritual care is the chaplain’s remit’ against ‘spiritual care is everyone’s business’.

**Table 3.** Responses by continent to others doing spiritual care and feelings about deployment.

				No	Yes
				n	n
*Did staff other than the chaplain do spiritual care?*			Australia	68	80
			Europe	233	192
			North America	250	266
*Did you feel that you could have been better deployed during the pandemic?*			Australia	118	23
			Europe	288	129
			North America	362	109

Cells with less than ten individuals are labelled <10 to lessen the risk identifying individuals.

### Did You Feel That You Could Have Been Better Deployed During the Pandemic?

Table 3 also shows that the majority of respondents felt they could have been better deployed during the pandemic. Free text comments to expand on this were given by 1260 participants, and the most frequently mentioned topic was ‘staff’ (n = 51), usually in the context of how chaplains could have been better used to support them. The comments as a whole covered a wide range of suggestions for improvement, often mentioning lack of engagement by management, poor organisation and poor communication. The follow up question was: *Did your organisation understand the contribution you could offer to COVID-19 patients?*

The majority (N = 911) said yes but 240 said no, with 120 elaborating by ticking ‘other’ and adding free text. In summary, a substantial minority (n = 360) reported feeling misunderstood.

### How Clear Was Your Role to You?

Participants were asked to reflect on each month since January. The general consensus remained somewhere close to neutral (neither clear or unclear) throughout, with clarity dipping in Europe and North America in March, before recovering slightly in April and May.

This pattern is consistent with free text comments suggesting role clarity fluctuated over time. It is also notable that the earliest measure (January), did *not* indicate a higher level of clarity in the role. In other words, the baseline position about clarity of role for the average chaplain *prior to the pandemic* was neither clear or unclear.

### How Have You Taken Care of Yourself Since the Beginning of the Pandemic?

**Figure 4.**
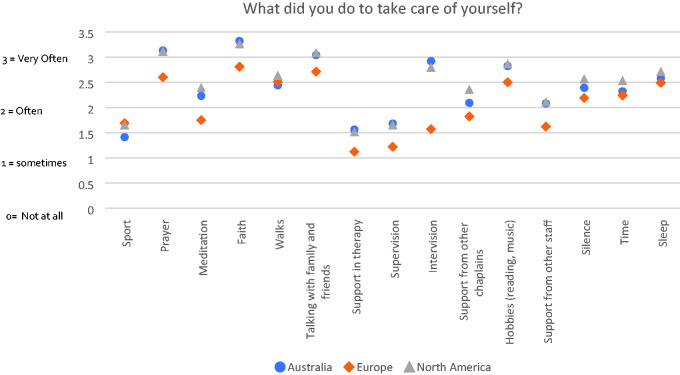
Responses by continent to a series of suggested ways chaplains took care of themselves.

This question was designed to find out what respondents found useful in relation to self-care. It entailed Likert style responses from zero (not at all) to 4 (All the time) to a series of suggestions such as sport, meditation, prayer, formal support structures and time, for example. Figure 4 summarises the mean scores for these examples by continent. In general, faith, prayer and the support of friends were the most frequently adopted strategies, with hobbies also outranking more formal types of support. It is interesting to note that Europe scored lowest of the three continents in 13 out of 15 examples.

### Which (If Any) of the Following Issues Had the Greatest Impact on You During the Pandemic?

**Figure 5.**
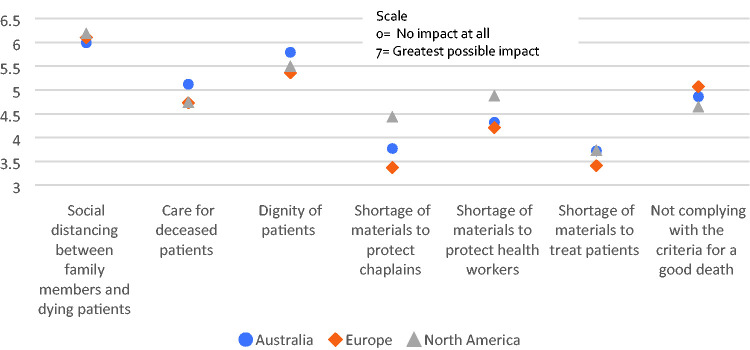
The impact of various aspects during the pandemic, by continent.

This question entailed a list of issues likely to have impacted chaplains, followed by a rating scale from 1 to 7, with 7 indicating the greatest impact, and 1 the least important. A total of 1245 participants completed this section and mean responses are illustrated according to continent in Figure 5. In summary, social distancing had the greatest impact on chaplains around the world, followed by concerns for the dignity of patients. Whilst all were clearly concerned about practical issues such as shortages of materials, it was those aspects impacting on their ability to help others that had the greatest impact.

### Was Your Professional Association and Faith/Belief Group Supportive?

This final question about support was followed by a series of examples, such as ‘*by organising special webinars*’, or ‘*by contacting you personally’*. Responses showed that personal contact was the least often experienced method of support and that emails or online support groups were usually the main source of support for all. This result also suggested that European colleagues may not have experienced the levels of formal organisational support experienced by their American and Australian counterparts.

## Discussion

The results support the picture painted in the introduction: many chaplains from countries around the world felt valued and understood by their employing organisations, adapted to using technology for communicating where necessary, got the right support from their professional associations, knew what to do to look after themselves and were very clear about their place in the healthcare team both before and during the pandemic. At the same time, a substantial proportion experienced the opposite.

All the respondents experienced *an* impact. Nearly all reported changes in work conditions. Social distancing had a substantial impact on chaplains in relation to reported barriers to them doing their job, with the dignity of patients being their biggest concern (Figure 5). The upheaval to working conditions that followed exacerbated enduring problems pertaining to professional identity, leadership and status (Busfield, 2020). For example, a majority stated they could have been better deployed, and a substantial minority suggested their organisations did not understand their role. The free text explanation below, from a single chaplain, encapsulates this broad theme; mourning the absence of chaplain leadership and feeling excluded.

1. *Chaplain leadership should have been at the executive leadership table during discussions of how to manage the pandemic, how to address patient isolation, family anxiety as a result of absence from their hospitalized loved ones.*
2. *Although we say that chaplains are integral team members, chaplains were not part of the discussions about both COVID-19 and non-COVID-19 patient care management at the outset. This should have happened.*
3. *Chaplains should have been involved in more and direct patient care throughout the pandemic…The attitude of leadership and nursing seemed to be that EVERYONE besides doctors and nurses would spread COVID-19, so everyone besides doctors and nurses must be excluded from encountering COVID-19 patients.* (N. America, male, 54)

**Figure 6.**
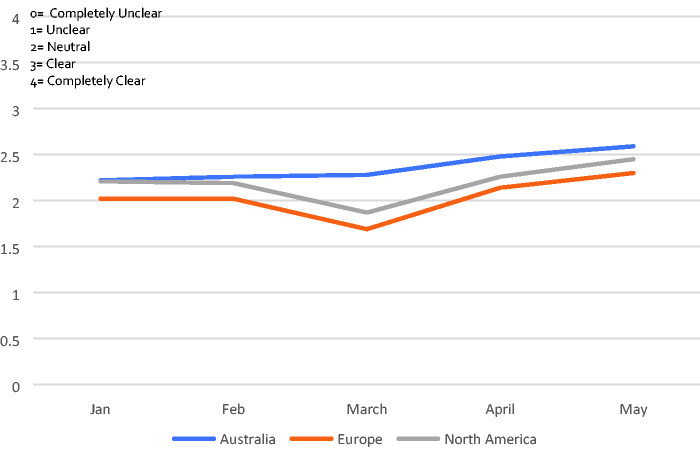
Clarity of role throughout pandemic by continent.

Subsequent papers in this series will examine the free text comments in depth. It is sufficient to point out that for some, instead of being considered an essential employee and valued colleague, many chaplains were instead seen as little more than an infection risk. Many pointed to the lack of chaplain leadership as the root cause of the problem, but very few acknowledged that ‘leadership is everyone’s business’ (Kouzes & Posner, 2017). This lack of confidence was evident elsewhere. For example, the average response to the question ‘*how clear was your role to you*’ was consistent both pre and post pandemic at a neutral point between ‘unclear’ and ‘clear’ (Figure 6). This meant that, on average, chaplains were not clear about what their role was *before* the pandemic. It follows that it would be unreasonable to expect managers and colleagues to be clear about how best to deploy chaplains when they are not clear themselves.

This is a professional issue. Recall the final column in Table 2 described how many chaplains were associated with professional associations. Although more prevalent in Australia, it showed that across the world approximately one in five chaplains was not associated with a professional association. It is very difficult to think of another health profession whose members are not subject to an agreed code of practice as authorised through their professional body.

This is important because one of the key purposes of the survey was to identify patterns in the data to generate hypotheses for further exploration, and it appears that lacking professional association could be a barrier to a healthier future for healthcare chaplaincy. For example, the survey showed that the frequency of supervision and/or intervision during the pandemic remained reasonably high in all three continents. Whilst this was unsurprising, it was noteworthy that European chaplains appeared to score lower on all measures of support than their Australian and US colleagues. Given that Europe also scored lower on 13 of 15 of the wellbeing measures shown in Figure 4, it may be that lack of professional status could account for this. There is primary evidence that Scottish chaplains registered with their professional association were more likely to feel supported than those not registered (Snowden et al., 2020). It follows that chaplains associated with professional associations may therefore actually *be* better supported.

To test this in this dataset, an independent-samples t-test was run to determine if there were differences in regularity of supervision between those associated with a professional association and those not. Those in a professional association reported more regular supervision (2.67 ± 0.6) than those not in a professional association (2.47 ± 0.03), a statistically significant difference of 0.2 (95% CI, 0.07 to 0.34), t(462) = 2.962, p = .003. In other words, across this international sample of chaplains, there was a significant relationship between being in a professional association and the amount of formal support received. Those affiliated with professional associations were also significantly more experienced in terms of years in the job (p > 0.001), and had higher education levels (p = 0.002) than their non-professional colleagues.

It is not possible to infer causality, but the evidence is mounting to suggest that being a part of a professional association is a fundamental good, even though most chaplains found faith, prayer and the support of friends and hobbies to be more useful than more formal types of support. The issue of professional status is closely related. More than half of the respondents reported seeing others doing spiritual care, and whilst this was welcomed by most, there was a vocal minority that felt others were encroaching on professional chaplain territory. This is consistent with Taylor and Li (2020), who found a considerable range of views when examining collaboration between chaplains and nurses. Taylor and Li found the best relationships between chaplains and nurses existed where they both worked regularly on the same unit and therefore had a clearer idea of each other’s roles and expertise. In these areas the chaplains were also much more likely to support the spiritual caregiving efforts of the nurses, whom they regarded as spiritual ‘generalists’, as opposed to themselves as spiritual ‘specialists’ (Taylor and Li, 2020).

The relationships between healthcare colleagues and chaplains is especially important because a lot of chaplaincy care was directed towards staff before Covid-19 (Fitchett, 2017; Liberman et al., 2020), and the survey data showed that caring for staff became an even more substantial aspect of their work during the pandemic. There is wider evidence that providing spiritual support to staff has been especially important during Covid-19. For example, in the enforced absence of the chaplain, some medics tried to deliver spiritual care as best they could (Sherwood, 2020). The same medics subsequently required the support of chaplains, to help them try to come to terms with their inability to save people. Just before Covid, Purvis et al. (2019) showed that healthcare staff were supportive of chaplain contributions to care, yet to date this hasn’t quite translated into others regarding them as part of the healthcare team. Perhaps in some settings Covid-19 has changed that, but the survey shows on the whole that a substantial proportion of chaplains remain outsiders. If they want to belong, they may need to reflect on their professional status in general.

## Limitations

Shortage of space necessitated summarising the survey data, and a different author may have selected different survey items to highlight. The usual limitations with self-report survey data apply too, such as recall problems and recency bias (Olson, 2014). Because we used our networks to sample as widely as possible, the actual response rate was unknown, so generalisability is compromised (Leeper, 2019). A further limitation was the absence of deeper analysis of many of the differences or relationships noted. For example, almost 50% Australian chaplains were not affiliated with professional associations, whereas the proportion was closer to 20% for North America and Europe (Table 2). This was not taken into consideration in the professionalism analysis. Likewise, American chaplains were educated to higher level on average by comparison to both Europe and Australia, although there are enduring arguments about whether it is possible to compare education levels across different countries and systems.

Nevertheless, the final sample was large, and all respondents had experienced a reaction to Covid-19, so in that regard there were more similarities than differences between the chaplains surveyed here.

## Conclusion

This international survey of healthcare chaplains generated considerable data. Every chaplain surveyed, regardless of age, gender, religion, or country, experienced a change in the way they worked as a function of the global Covid-19 pandemic. The impact of having to distance themselves from patients was the most problematic aspect for all chaplains. Whilst clarity improved over time, and chaplains engaged with technology to support their patients and staff, most respondents weren’t clear about their role during the pandemic. Perhaps more surprisingly, the survey showed that chaplains weren’t clear about their role *before* the pandemic either.

In conclusion, chaplaincy wasn’t ready for the pandemic. The same was likely true of every other health profession, to a degree, but the emergency phase of the pandemic shone a bright light on healthcare professionals to identify those essential, and those not. The vast majority of chaplains found themselves in the latter category, blaming the lack of senior chaplain leadership for their fate. Yet leadership is everyone’s business. Chaplains *are* essential, but are alone amongst their international healthcare colleagues in *not* having compulsory professional registration. One of the consequences of this is that there is no way of speaking for chaplaincy as a unified profession with clear vision and identity. Until this changes, chaplains will continue to be undervalued and misunderstood, and the people who suffer most from this are the patients they serve so uniquely.

## Note

1 * or equivalent. For example in Australia, specialists in spiritual care are called Pastoral Care Workers
